# Shape-position perceptive fusion electronic skin with autonomous learning for gesture interaction

**DOI:** 10.1038/s41378-024-00739-9

**Published:** 2024-07-22

**Authors:** Qian Wang, Mingming Li, Pingping Guo, Liang Gao, Ling Weng, Wenmei Huang

**Affiliations:** 1 State Key Laboratory of Reliability and Intelligence of Electrical Equipment, Tianjin, China; 2 Key Laboratory of Electromagnetic Field and Electrical Apparatus Reliability of Hebei Province, School of Electrical Engineering, Hebei University of Technology, Tianjin, 300130 China

**Keywords:** Electrical and electronic engineering, Sensors

## Abstract

Wearable devices, such as data gloves and electronic skins, can perceive human instructions, behaviors and even emotions by tracking a hand's motion, with the help of knowledge learning. The shape or position single-mode sensor in such devices often lacks comprehensive information to perceive interactive gestures. Meanwhile, the limited computing power of wearable applications restricts the multimode fusion of different sensing data and the deployment of deep learning networks. We propose a perceptive fusion electronic skin (PFES) with a bioinspired hierarchical structure that utilizes the magnetization state of a magnetostrictive alloy film to be sensitive to external strain or magnetic field. Installed at the joints of a hand, the PFES realizes perception of curvature (joint shape) and magnetism (joint position) information by mapping corresponding signals to the two-directional continuous distribution such that the two edges represent the contributions of curvature radius and magnetic field, respectively. By autonomously selecting knowledge closer to the user's hand movement characteristics, the reinforced knowledge distillation method is developed to learn and compress a teacher model for rapid deployment on wearable devices. The PFES integrating the autonomous learning algorithm can fuse curvature-magnetism dual information, ultimately achieving human machine interaction with gesture recognition and haptic feedback for cross-space perception and manipulation.

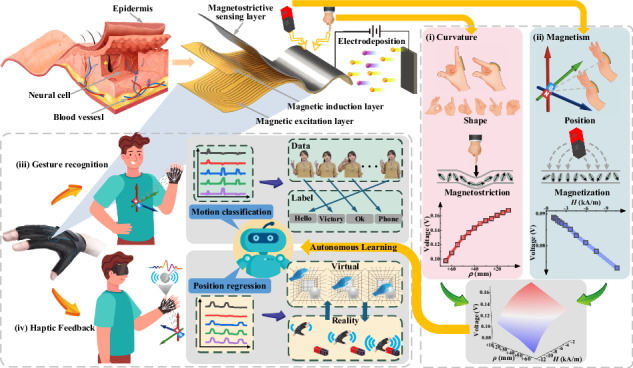

## Introduction

Hand gestures are considered more natural and user-friendly interactions for virtual reality (VR)^1^, augmented reality (AR) and other human‒machine interaction (HMI) systems^2–4^. Wearable devices^5,6^ consisting of electronic skins (e-skin)^7^ can perceive human instructions, behaviors and even emotions by tracking hand motion with the help of deep learning^8,9^; in contrast, visual images/videos^10^ and brain-computer interfaces^11^ are affected by environmental interference, such as blocked objects and physical conditions.

Standalone bendable and stretchable sensing platforms powered by human motion^12–14^, biofuel^15,16^ or radio frequency (RF)^17^ can be integrated into wearable devices, clothing or accessories to unobtrusively and accurately monitor human movement^18,19^. Multiple fabrication methods, including printing^20^, magnetron sputtering^21^, and electrodeposition^22^^,^ have been widely explored to integrate micro/nanoscale film devices prepared on planar growth substrates onto the surface of the target substrate for the preparation of small functional devices. They can be combined with transfer print^23^ or assembly^24^ methods to allow precise deposition of electronic components and interconnections on curved or irregular surfaces for device integration on 3D surfaces conforming to the human body structure^25^. However, the abovementioned sensors are typically limited in their ability to measure the shape of the user’s finger joints, resulting in a lack of position information and creating an intention gap between gestures and truth labels^26,27^. For example, the shape of the hand in which the thumb is stretched out in a one-handed fist, with the thumb pointing up (Like) and down (No-like) having opposite meanings, is indistinguishable to these sensors that detect only finger movements. The magnetosensitive e-skins^28^ based on spin valves are able to improve magnetic perception^29^ for body position tracking^30^ and touchless object operation^31,32^. However, working in isolation still faces a peculiar set of problems related to in-plane magnetic sensing and nondirect sensory feedback^33^.

Multimode fusion^34,35^ fills this gap and significantly improves classification performance^15,36^ through the use of accelerometer sensors^37–39^ and contact forces^40^. In addition, complex multimodal fusion algorithms and hardware integration increase power consumption and size^41^, in contrast to the original aim of designing wearable sensors. Sensors that can respond to stress and magnetic fields simultaneously^42^ have the potential to be used in VR and AR for sensing the motion of the user’s hand. Currently, their sensitive components are composed of polymers with magnetic particles dispersed within them. When external stress or magnetic fields are applied to this type of sensor, magnetic particles will move and lead to changes in the magnetoresistance^42^, resistance^43–46^, capacitance^47^, electric friction^48^, and displacement reaction voltage^49^, thus achieving normal stress and proximity sensing. Nevertheless, magnetic particles cannot distinguish the different directions of the forces or magnetic fields, making sensing microstructures ineffective in complex wearable applications, such as tension, compression, bending and in-plane magnetic fields^50^. This switching and identification of force-magnetism dual modes are inconvenient for signal postprocessing and, in turn, slow the response time.

**Fig. 1:**
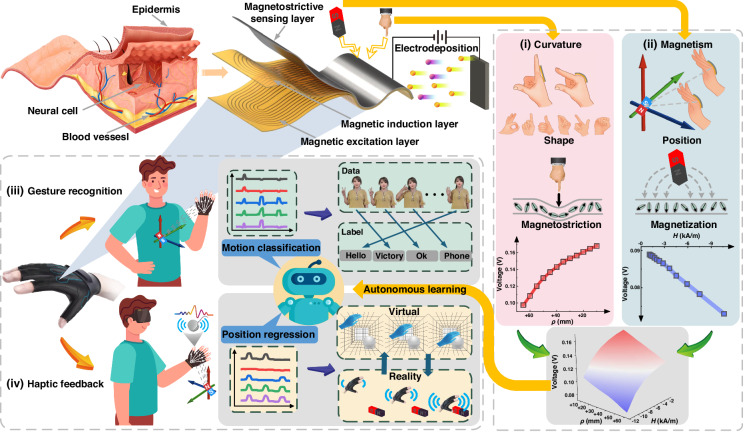
The PFES has a bioinspired hierarchical structure consisting of a magnetostrictive sensing (MS) layer, a magnetic induction (MI) layer and a magnetic excitation (ME) layer, to imitate the human somatosensory system. Based on the magnetostriction and magnetization effect of the MS layer, the PFES outputs electrical signals through the MI layer under the excitation of the ME layer, realizes fusion of curvature (i) and magnetism (ii) information by mapping corresponding signals to the two-directional continuous distribution such that the two edges represent force and magnetic contribution. Installed at the joint of a hand, the PFES can perceive hand shape and position simultaneously, ultimately achieving human machine interaction with gesture recognition (iii) and haptic feedback (iv) for cross-space perception and manipulation based on the autonomous learning algorithm

This study presents a fast-response, mechanically durable, low-cost, and light-thin electronic skin that perceives curvature and magnetism information by mapping corresponding signals to a two-directional continuous distribution whose ends represent force and magnetic contributions, respectively (Fig. 1). The developed perceptive fusion e-skin (PFES) has a bioinspired hierarchical structure comprising a magnetostrictive sensing (MS) layer, magnetic induction (MI) layer and magnetic excitation (ME) layer. The distribution of magnetic domains of the MS layer is subject to curvature (i) and magnetism (ii) based on magnetostriction and magnetization effects and produces opposite responses accordingly under the excitation of the ME layer. These magnetization state variations are then converted into analyzable electrical signals by the population response of the pickup coils in the MI layer, which exhibits high vertical coupling and low in-plane interference. After synthesizing the force and magnetic component, the electrical signal of the PFES output is positively related to the curvature and negatively related to the magnetic field for characterizing the bend and position. This comprehensive biperceptive information from a single PFES enables the recognition of motion features from low-dimensional data, thereby reducing the processing costs of signals and computations.

The PFES is installed on the hand joints to perceive the bending and magnetic field fusion signals and to collect the sensing data during the gesture interaction. Deep learning models are built to quickly recognize and accurately extract the shape and position features of a hand from sensor data, enabling gesture recognition (iii) and haptic feedback (iv) through motion classification and position regression. Although large-scale deep models can be collected and trained on a large amount of user data to obtain state-of-the-art results, these models need to be compressed to be deployed in wearable devices requiring local signal processing and a miniaturized form factor, and the repetitive data collection for each user increases the time cost of deployment. Therefore, we developed Deep Reinforcement Learning-based Knowledge Distillation (DRL-KD) and constructed multiple pretrained models to act as teachers, compressing them into deployable student models. The dynamic assignment of weights to teacher models based on a loss function enables student models to autonomously select the teacher models and their associated knowledge that are more closely aligned with the user’s motion characteristics. This approach avoids the impact of the complexity of the training samples and differences in model capabilities on the performance of the student models. DRL-KD enables rapid deployment of gesture interaction tasks between real space and virtual space by training student models w ith few-shot samples.

## Results

### The PFES for curvature radius and magnetic field perception

The Co-Fe film, as a magnetostrictive material, can be utilized in the MS layer of the PFES due to its large magnetostrictive coefficient and excellent soft magnetic properties under low fields^51^. Copper or titanium foil, used as a cathode and electrodeposited substrate, was cleaned with acetone and rinsed with deionized water. The anode of the platinum strip was fixed parallel to and centered on the cathodes at a distance of 3 cm. The composition of the baths included 0.3 mol/L H_3_BO_3_ (buffer), 0.3 mol/L NaCl (improve conductivity), 0.4 mol/L ascorbic acid (antioxidant), 0.4 mol/L glycine (complexing agent), and 0.3 mol/L sodium citrate (complexing agent). The electrolyte pH was controlled at 2.5 with 10% H_2_SO_4_. Mild temperature (50 °C) and stirring conditions were applied to enhance the mass transport of metal from the bulk electrolyte toward the electrode surface.

We chose Co_70_Fe_30_ as the magnetostrictive sensitive material with a main salt concentration of 0.08 mol/L FeSO_4_ and 0.3 mol/L CoSO_4_ because of its excellent magnetostriction, permeability and toughness^52^. The depositions at constant current were carried out at 0.035 A/cm^2^ for 40 min. The current for plating each sample was kept constant for 40 min, leading to a film thickness of approximately 20 μm. Following electrophoretic deposition, the samples were rinsed with deionized water, dried with hot air, and obtained from Ti substrates by mechanical action. The nominal composition and crystal structure of the prepared bimetallic films were determined by energy dispersive spectroscopy (EDS) and X-ray diffraction (XRD). The experimental results show that the nominal composition of the film is Co_70_Fe_30_, and it has a body-centered cubic structure (PDF 50-0795) and a strong 110 peak.

**Fig. 2:**
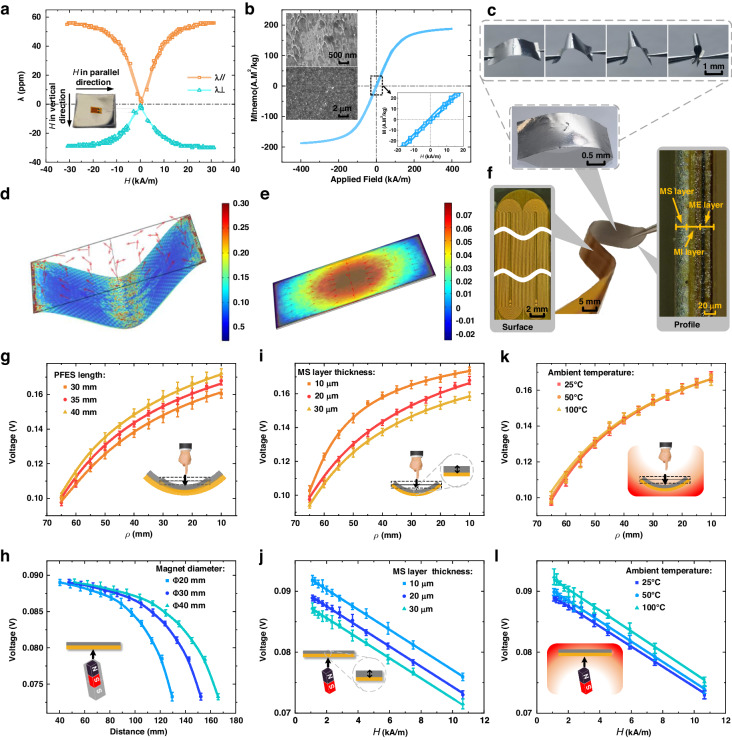
The properties of electrodeposited magnetostrictive film and the PFES design. **a** Magnetostrictive curve. **b** Magnetization hysteresis loops. Insets show the surface and fracture SEM images of Co_70_Fe_30_ film. **c** Fabrication of the electrodeposited Co_70_Fe_30_ films: original and bending state. **d** The direction (red arrows) and magnitude (color mapping) of magnetization under bending strains. **e** The direction (red arrows) and magnitude (color mapping) of magnetization under permanent magnet. **f** The integral, profile and back picture of the PFES. **g** The effect of different sensor lengths (30 mm, 35 mm and 40 mm) on the PFES output voltage amplitude at curvature radius. **h** The effect of different magnets diameter (Φ20*5 mm, Φ30*5 mm and Φ40*5 mm) on the PFES output voltage amplitude at magnet distances. Comparison of the voltage-curvature radius curves (**i**) and voltage-magnetic field curves (**j**) with different thicknesses of MS layer. Comparison of the voltage-curvature radius curves (**k**) and voltage- magnetic field curves (**l**) with different ambient temperatures

The results are shown in Fig. 2a, and the inset shows that the strain gauge attached to the specimen surface is parallel or perpendicular to the field. The electrodeposited Co_70_Fe_30_ alloy film has good magnetostrictivity. The magnetization hysteresis loops were measured by a physical property measurement system (PPMS) at room temperature. The magnetization loops of the film under a vertical magnetic field are shown in Fig. 2b, which shows the sensitivity under a low magnetic field and favors the induced magnetic field of the MS layer. The surface and fracture morphology of the Co_70_Fe_30_ films show that there is no chapping on the surface and that the fracture is smooth and flat, as shown in the inset. The Co-Fe film prepared by electrodeposition has a distinct metallic luster and exhibits excellent flexibility under the action of external forces, as shown in Fig. 2c.

Based on the material’s sensitivity to strain and magnetic field, the structure of the PFES was designed by analyzing the response of the film under bending and magnetic field. COMSOL Multiphysics was employed to visualize the magnetization distributions during force and magnetic field interactions, as schematically illustrated in Fig. 2d and e. When curvature or a magnetic field generated by bending strains or a magnet is applied at the center of the film along the normal direction, the changes in the magnetization state obviously change the magnitude of the field at the center of the film. Accordingly, placing the coils at the center of the film allows for maximum collection of magnetization changes in the vertical direction.

Figure 2f shows a detailed picture of the PFES fabricated by high-performance MEMS (microelectromechanical system) technology. Both the ME layer and MI layer are manufactured from flexible printed circuits with a layer thickness of 40 µm. The substrate material (polyimide) is bonded to the etched copper layer through epoxy resin and lamination methods, and the thicknesses of the substrate and copper layer are 12.5 µm and 18 µm, respectively. The ME layer, which contains one pair of planar coils, generates an alternating magnetic field on the MS layer through alternating current passing through the coils. The MI layer is made up of another pair of pick-up coils connected in a subtractive series to cancel out the transformer effect in the coils, resulting in a net magnetic flux that is proportional to the strength of the external curvature and magnetic field on the MS layer^53^. The turn ratio of the exciting coils to the pick-up coils is 1:1. The routing width and clearance of the flexible printed circuit (FPC) are both 70 µm. The MS layer is a magnetostrictive Co_70_Fe_30_ film (thickness of 20 µm) electrodeposited on top of the copper layer. Due to the small impedance angle, the PFES can be directly excited without a matching circuit. Considering the current load and power consumption of the coil, the exciting coils are operated with 10 mA and 5 kHz current. Based on the impedance of the coil, we can calculate that the average power of the PFES is less than 25 mW.

The performance of the PFES was verified at different curvature radii *ρ* and magnetic fields *H* by loading bending strains (3-point bend fixture) or permanent magnets (NdFeB N35 Φ30*5 mm) (see Materials and Methods). The output voltage of the MI layer is amplified 200 times by a voltage preamplifier and subsequently analyzed using a lock-in amplifier, with the data monitored on a PC. If the electrodeposited MS layer is defined as the front side and the substrate is defined as the back side, the symbols of the force and magnetic field applied to the front side of the sensor are positive, and vice versa.

First, based on the response shown in the simulation, the PFES can evaluate the bending strain and magnetic field by the output amplitude, taking into account the effect of the sensor and magnet size. Figure 2g shows that the PFES output is related to the curvature radius at different PFES lengths ranging from 30 to 40 mm. Extending the PFES length is beneficial for improving the sensitivity because longer PFESs obtain more uniform material strain at the same radius of curvature. However, we chose 35 mm as the device size to match the size of the finger and joint. Figure 2h shows the effect of different magnets (NdFeB N35) of Φ20 × 5 mm, Φ30 × 5 mm and Φ40 × 5 mm on the PFES output at different distances to evaluate the effect of PFES position detection. Larger magnets provide a stronger magnetic field that can be used in applications with longer distances.

Second, the thickness of the MS layer also has a significant effect on the sensitivity. Co-Fe films with thicknesses of 10 μm, 20 μm, and 30 μm were prepared by electrodeposition. For devices with different MS layer thicknesses, the variations in the output voltage with curvature radius ranges of 10–65 mm and magnetic field ranges of 1–11 kA/m are shown in Fig. 2i and 2j, respectively. The results show that the sensitivity of the device reaches its maximum value when the MS layer film thickness is 20 μm. As the thickness of the force magnetically sensitive film increases, the coupling effect becomes stronger, which increases the sensitivity, while the crystal brittleness also increases, which decreases the sensitivity. This trade-off effect is most favorable when the thickness is 20 μm. Finally, the effect of different temperatures on the FPES output was evaluated. Figure 2k and l show the dependence of the output signal on *ρ* and *H* at different temperatures by integrating a high-temperature system (furnaces and control software) into the test platform. The measurement error is less than 4.2% from 25 °C to 100 °C, which verifies that the sensor has good reliability at high temperatures.

**Fig. 3:**
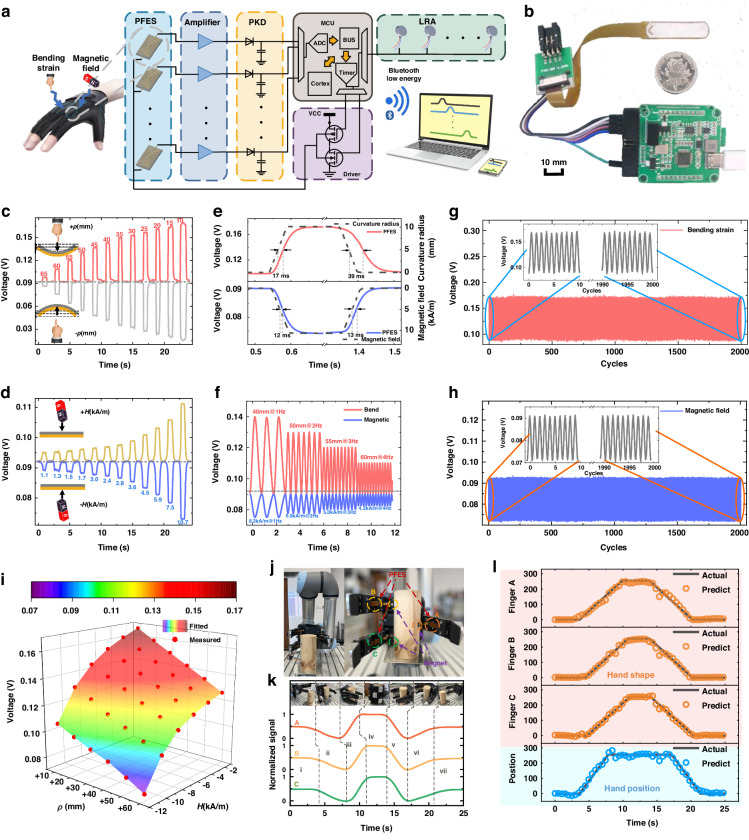
The output characteristic of the sensor system based on the PFES. **a** The real-time control circuit handles exciting, pickup and vibrative signals that support the PFES and are sent to the PC. **b** The images for the assembled PFES with the wires and control circuit board. **c** The PFES output voltage under ρ of 10–65 mm. **d** The PFES output voltage under H of 1–11 kA/m. **e** Response time and recovery time of the sensor at ρ of 10 mm and H of 11 kA/m. **f** Output voltage waveform under dynamic force and magnetic field at 1–4 Hz. Mechanical durability test for up to 2000 load-release cycles at curvature radius (**g**) and magnetic field (**h**). Inset: the voltage signals generated for the initial 10 s and the final 10 s. **i** The measured (red dot) and fitted (color fill surface) output voltage amplitude under different ρ and H. **j** Assembly drawing of the PFES and magnet on the robotic hand and object. **k** Change of the sensor output when the finger bearing the sensor approaches, grasps, loosens and retreats from the magnet-decorated object. **l** The predicted values output by deep learning are generally consistent with the actual values, proving that the sensor can assess the shape and position of the hand

The circuit diagram in Fig. 3a illustrates the real-time control circuit generating the exciting, pickup and vibrative signals that support the PFES and are sent to a PC for analysis. The output voltage generated by the MI layer first passes through the half-wave amplifier, then is transmitted to the peak detection (PKD) voltage amplifier, and finally is converted into a 12-bit digital signal by an analog-to-digital converter (ADC). The linear resonance actuator (LRA) is driven by PWM and a haptic driver. A 100% input duty cycle provides full vibration intensity, while a 0–50% input duty cycle results in no vibration intensity and always enforces braking. The dual-output 3 A MOSFET driver provides a ± 10 V square wave as the excitation current for the PFES. The control circuit can transmit the digital signal of several PFESs via Bluetooth to the computer for further analysis. A biperceptive sensor system consisting of control circuits and a PFES can be used to obtain the signal of the curvature radius and magnetic field, as shown in Fig. 3b.

Figure 3c depicts the output amplitude of the sensor under a *ρ* of 10–65 mm along two opposite directions by loading different deflections to the PFES. The PFES output shows strong nonlinearity under large strain, which is caused by the hysteresis characteristic of magnetostrictive materials. Figure 3d illustrates the output amplitude of the PFES under *H* of 1–11 kA/m along two opposite directions resulting from different distances from the magnet. Due to the distance of the magnet from the PFES and the small applied magnetic field, the PFES output appears to be linear over the magnetic field range. The response curves of the experimental measurements are symmetrical in two opposite directions. The maximal sensitivities of the PFES are 22 mV/mm within a curvature radius of 10–65 mm and 1.78 mV/(kA/m) within a magnetic field of 1–11 kA/m. The experimental results indicate that the PFES exhibits good static characteristics, and its output shows consistent behavior in both directions, enabling flexible design of application scenarios.

Figure 3e characterizes the loading and unloading output amplitude under a *ρ* of 65 mm and *H* of 11 kA/m for testing the hysteresis of the PFES. The maximum response and recovery times of the PFES were 17 ms and 39 ms, respectively. Figure 3f shows the dynamic output characteristics of the PFES under force and magnetic fields with frequencies ranging from 1 to 4 Hz. The changes in the sensitivity are less than 1.6% at 1–4 Hz dynamic bending strains or magnetic fields, which indicates good dynamic stability. The signal duration of the sensor changes with the curvature radius and magnetic field, as shown in Fig. 2g and 2h. Under periodic stimulation (under a *ρ* of 65 mm and *H* of 11 kA/m) at 4 Hz, the output voltage of the sensor does not decrease significantly after 2000 cycles of continuous loading, indicating a good voltage signal duration.

Figure 3i shows the measured (red dots) and fitted (color-filled surfaces) output voltages when *ρ* and *H* are loaded simultaneously in the range of curvature radius +10 to +65 mm (loading above the PFES) and magnetic field −1 to −11 kA/m (loading below the PFES). After synthesizing the force and magnetic component, the electrical signal of the PFES output is mapped to a continuous surface to merge the perceptions of curvature and magnetic field. Due to the superposition of two responses with different positive curvature radii and negative magnetic fields, the PFES output shows nonlinearity but has monotonicity in the measuring range. The output signal is positively related to the curvature, and the magnetic field negatively related to the interaction effect of bending and positioning. We note that the sensing responses might be present in overlapping regions, causing confusion about the curvature and magnetic information if the direction of the applied curvature or magnetic field is changed.

By designing interaction experiments between the manipulator and the object and combining sensor fusion data and deep learning methods, inference of the hand shape (bending attitude) and position (spatial distance) information can be achieved. Figure 3j shows a schematic diagram of the three-finger manipulator and grasped object, where the magnetic beacons and the sensor system are installed on the grasped object and the three-finger manipulator, respectively, for comprehensive sensing and fusion of finger attitude and object position. The flexible sensor is closely attached to the inside of the finger joint of the manipulator through adhesive tape, which does not prevent the manipulator from performing any action and ensures that the flexibility of the manipulator is not affected. The sensor outputs a positive signal for the characteristic curvature when subjected to bending. The magnet is fixed on the surface of the object and points at the sensor, which outputs a negative signal by adjusting the field of the magnetic beacons in terms of polarity and strength. The three-finger manipulator with the sensor grasps the object with magnetic beacons, the displacement and grasping speed of the manipulator were set to 50/150 and 30/150, respectively, and the manipulator motor encoder data and sensor output voltage were collected.

The sensor system is calibrated using the maximum and minimum output signal values of the sensor unit, mapping the signals to a range of 0 to 1. Figure 3k shows the variation in the sensor output voltage while approaching the object, grasping it, loosening the grasp of the object and finally retracting the finger from the object. A decrease in the output signal corresponds to a decrease in the distance from the object in stage i, when the manipulator approaches the object and fingers unbend. In stage ii, the object enters the grasp range of the manipulator, and the finger joint is driven to grasp the object; meanwhile, the signal is less than 0.5 and further decreases. The minimum signal is acquired when the finger joint touches the object surface and then enters stage iii. During the grasping process, the signal rapidly increases and becomes positive due to the curvature of the joints. As soon as the manipulator grasps the object stably, the output remains constant with the maximum joint curvature at stage vi. The output signal is opposite to that of the approaching and grasping process when the manipulator loosens its grasp and retreats from the object at stages v, vi, and vii.

Considering the temporal correlations inherent in manipulator movements, we leverage long short-term memory (LSTM) networks to construct a learning model for better capturing long-term dependencies in time series data. Utilizing data from multiple repeated experiments, a deep learning model was trained to predict the hand shape and position. Figure 3l illustrates that the sensor-based model predictions are in general accordance with the measured ground truth, and the deviation between them is less than 15%. Experimentally, it is demonstrated that the sensor output signal fuses the curvature radius and magnetic field, which can reflect the hand shape and position information through scene design and deep learning.

### Distilling student model to recognize and interpret gestures via autonomous learning

**Fig. 4:**
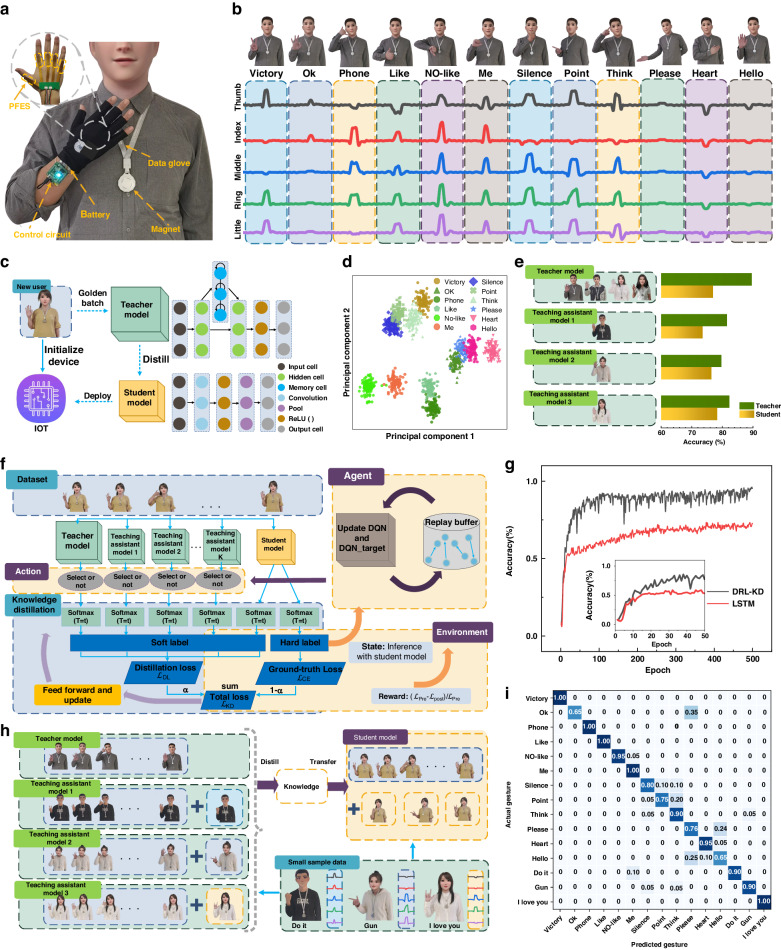
Reinforced knowledge distillation (DRL-KD) principle and experimental results. **a** Photograph shows human-computer interaction platform including the real-time control circuit, battery, magnet and the PFES in the data glove. **b** Photographs of confused gestures and the corresponding voltage profiles. **c** Knowledge distillation deploys deep learning models on small devices with limited resources for new users. The teacher and student models are LSTM and CNN, respectively. **d** Visualization of the two-dimensional features of the 12 motion vectors. **e** The accuracy of different magnitudes teacher networks and their distilled student networks. **f** The student model learns and compresses the different teacher models by knowledge distillation and a few-shot label dataset, to autonomously select knowledge closer to the user's characteristics by deep reinforcement learning. **g** Inference accuracy trends for gesture recognition with further training epochs: DRL-KD distilled student model (black line) by few-shot data and 3-layer LSTM model (red line) trained with all the data. **h** The student model can select a teaching assistant model to learn knowledge without involving the teacher network by the DRL-KD. **i** The confusion matrix of hand-motion signals for 12 gestures in a teacher model and additional 3 gestures in a teaching assistant model

The lightweight and curved PFES can comfortably accommodate human hands or a glove surface and is cost-effective for large-area applications of electronic skin. Figure 4a shows a human-computer interactive platform composed of a real-time control circuit, a battery (1500 mAh, 5 V, working for approximately 3–4 h), a magnet and a data glove. To ensure the independence of signals between each unit, PFESs are installed exclusively at the metacarpophalangeal joints, considering their association with motion. The PFES is fixed at the position of the corresponding user’s knuckles in the sewing interlayer of the glove. The magnetic beacon (NdFeB N35 Φ40*5 mm) is set on the user’s chest, and the opposite direction of the magnetization point at the PFES is used to output a negative signal. The PFES and the circuit components are integrated in the glove to construct a data glove. The PC Bluetooth connection is virtualized as a serial device, and the host computer is equipped with the MATLAB GUI.

To correct PFES output discrepancies resulting from varying wearing habits and manufacturing errors, a normalization process is employed during the dataset establishment phase. The signal is mapped to the range of 0 to 1 based on the maximum and minimum values of the PFES output. To demonstrate the data glove system, twelve motions were selected, which included common gestures and easily confused gestures. Traditional stress/bending sensors have difficulty recognizing gestures with finger joints of similar shape and different positions (directions); however, their meanings are completely different, such as ‘Like, NO-like, Me’, ‘Silence, Point, Think’, and ‘Please, Heart, Hello’. The corresponding voltage profiles of these gestures are shown in Fig. 4b.

The knowledge of the large-scale teacher model is transferred to the lightweight student network to achieve model compression and deployment in wearable devices through succession between the master and the apprentice, as shown in Fig. 4c. The teacher model is a pretrained model with 3-layer LSTM. The student model is a tested model with a 1-layer convolutional neural network (CNN). The teacher model is trained by the data collected from four participants. Each assistant teacher model and student model are trained and tested on the data collected from one participant. Each participant repeated every gesture 100 times to collect the data, which visualized the two-dimensional features of 12 motion vectors via principal component analysis (PCA), as shown in Fig. 4d.

To train the student model, the distillation temperature T is updated depending on the total loss ${{\mathscr{L}}}_{{\rm{KD}}}$ by feeding forward. ${{\mathscr{L}}}_{{\rm{KD}}}={\rm{\alpha }}{{\mathscr{L}}}_{{\rm{DL}}}+(1-{\rm{\alpha }}){{\mathscr{L}}}_{{\rm{CE}}}$, where α is a hyperparameter to balance ${{\mathscr{L}}}_{{\rm{DL}}}$ and ${{\mathscr{L}}}_{{\rm{CE}}}$ (α = 0.8 in this example). The distillation loss ${{\mathscr{L}}}_{{\rm{DL}}}$ is defined as the cross-entropy loss of predictions between the teacher model and the student model based on soft labels. The ground-truth loss ${{\mathscr{L}}}_{{\rm{CE}}}$ is defined as the cross-entropy loss between the hard label of the student model and the ground-truth label. The teacher models are trained by collecting data where 80% of the samples are used for training and 20% of the samples are used for testing. The corresponding student models are distilled through 3 samples in each gesture, and other samples are used for testing. Figure 4e shows that massive amounts of data and a large network structure help to build a stronger pretrained model. However, a stronger teacher model may not necessarily lead to a better student model due to the complexity of training examples and differences in student model capability.

To achieve good distillation, we adopt a value-based reinforcement learning method to choose teacher models. The agent matches the characteristics of the new user for different training instances and optimizes the performance of the student model. The compressed model, which involves transferring knowledge from multiple teachers to a single small student model, has been highly successful in the domain of natural language^54^ and video data^55^ processing. In contrast with gesture recognition tasks, pretrained models have large numbers of model parameters and training data. Recently developed methods^56^ have been designed to calculate the reward via the standard policy gradient method and require large amounts of sample data. Analogous to how a person purposefully chooses to learn teachers’ knowledge for completing tasks through the accumulation of practice and experience, deep reinforcement learning (DRL) with experiential replays learns from different teacher models via a few-shot label dataset. We select the dueling deep Q-network (Dueling DQN)^57^ as the learning agent to perceive the distillation state, where the dominance function enables faster identification of the correct action^58^.

The agent interacts with the environment and samples teachers to participate in the knowledge extraction step, as shown in Fig. 4f. The agent chooses between two possible actions (select or not) in each teacher model for the current instance. The inference with all teachers is used to calculate the distillation loss ${{\mathscr{L}}}_{{\rm{Pre}}}$. ${{\mathscr{L}}}_{{\rm{Post}}}$ is calculated via inference with selected teacher model ensembles after DRL optimization. We calculate the rate of change in the total loss ($({{\mathscr{L}}}_{{\rm{Pre}}}-{{\mathscr{L}}}_{{\rm{Post}}})/{{\mathscr{L}}}_{{\rm{Pre}}}$) as a reward. The sampled teacher’s output is integrated into a soft label to train the student model. The inference of the distilled student model is taken as environment states that summarize the characteristics of the input instances and the candidate teacher models. The loss function and current state are employed to update the DQN network and are stored in the replay buffer for uniform random sampling. In each iteration, the agent produces an action according to the DQN network and obtains the next state and reward in the environment. These elements (state, action, reward, next state) involved in DRL iterate during episodes until the performance of the student model converges. Figure 4g demonstrates our model’s ability to transfer knowledge to a student model for a new user compared to a traditional deep learning network trained using a large amount of data. Although possessing the same structure as the teacher model, the 3-layer LSTM model requires more than 300 training epochs to achieve 60% accuracy. The distilled model with our developed learning framework achieved more than 80% accuracy within 40 transfer training epochs using few-shot data (3 samples in each gesture).

In addition to the model deployment of new users, frequent registered new gestures are also common application scenarios. We can import new gestures contained in other user modes by the small sample learning abilities of knowledge distillation, as shown in Fig. 4h. To transfer knowledge to student models, other user models are distilled with teacher models as teaching assistant models. Based on the DRL-KD method, the distilled student model is used to classify 12 original gestures and 3 new gestures, and the performance of this model is evaluated by means of a confusion matrix, as shown in Fig. 4i. Each row of the matrix represents the test samples in an actual class, and each column represents a predicted class. The original 12 gestures achieved a classification accuracy exceeding 86.75%, and the three newly added gestures achieved an accuracy of 93.33%. The overall accuracy is 88.06%.

### Interaction with a magnetic field and virtual object by gesture control feedback

**Fig. 5:**
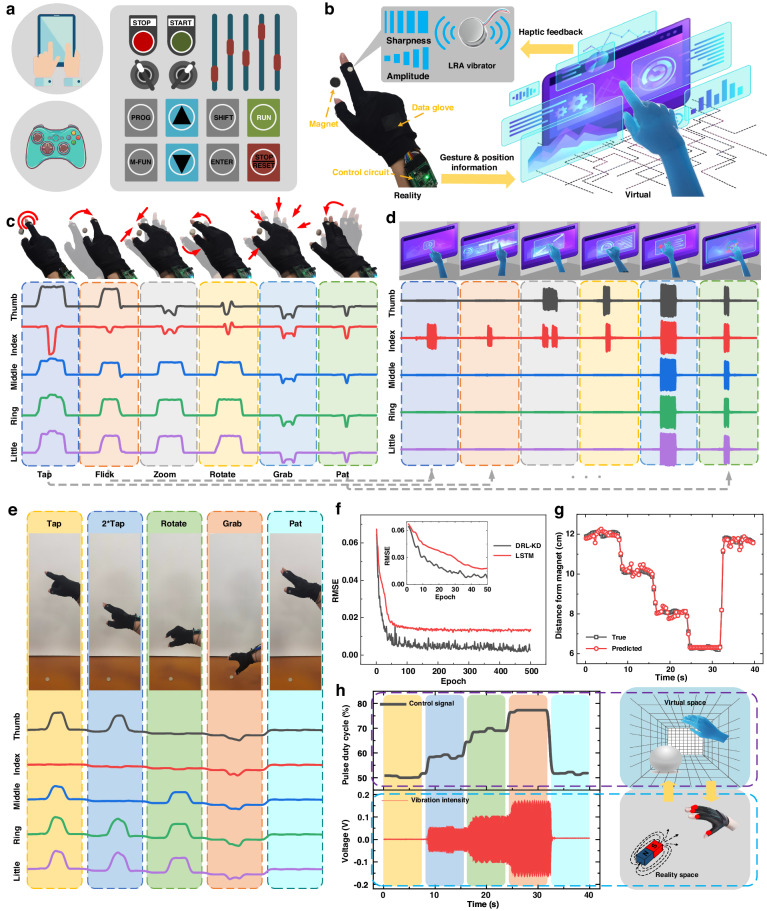
Virtual interactive operations and augmented feedback based on PFES for HMI. **a** Traditional HMI devices (e.g., touch screen, handles and control panels) provide intuitive and rapid feedback through vibration or sound-light alarm. **b** Illustration of the human-computer interactive platform integrating with LRA vibrators to achieve the cross-space communication of real and virtual information. **c** Output signal of gesture control by the interactive platform and magnet. **d** Real-time vibration intensity corresponding to control feedback. **e** Interactive operations at different distances with the magnet and the corresponding PFES signals. **f** Normalized RMSE trends for gesture recognition with further training epochs: DRL-KD distilled student model (black line) using few-shot data and 3-layer LSTM model (red line) trained with all the data. **g** The distance between the user's hand and the magnet: the predicted value output by the model and the real value obtained by the measurement are compared. **h** Control signal of the platform and vibration intensity collected by a PZT vibration sensor

In addition to machine acquisition of the advanced strain-magnetic dual-perception fusion capability, users need intuitive and rapid feedback similar to traditional input‒output devices (touch screen, handles and control panels) that provide vibration or sound-light alarms, as shown in Fig. 5a. We incorporate vibration sensations into the real sense to help individuals interact with virtual objects and sense the magnetic field. A human-computer interactive platform with haptic stimulation can simultaneously sense human movement and simulate human sensations to establish an immersive VR/AR experience through the amplitude and sharpness of haptic feedback. Based on the PFES, haptic feedback further enhances the connection between the physical and cyber worlds. The interactive platform with augmented feedback is realized by integrating the linear resonance actuator (LRA) onto the data glove, as illustrated in Fig. 5b. In this study, the PFES at the five joints recorded the movements of the human hand, and a magnetic beacon (NdFeB N35 Φ20*5 mm) was placed beneath the palm to simulate virtual objects. It allows the operator who virtually simulates real scenes to realize human-computer interaction in a more natural way, such as space location and gesture control.

Additionally, simultaneous multifinger sensing and feedback can be applied to virtual control panels in AR applications and nontouch gesture operations for wearable VR devices, such as tap and rotate buttons, flicks and zoom screens. The PFES on each finger can independently monitor finger motion for real-time acquisition and provide different spans of vibration feedback after receiving a nontouch magnetic signal, as shown in Fig. 5c and d. A magnet with low remanence is selected to better recognize the bend signal easily submerged by a magnetic field. When the output voltage of the PFES is below a set threshold, people perceive magnetic fields as driving the LRA and generating vibrations. When nontouch gestures operate above the magnet, augmented haptic feedback helps the user to master the action effect and relative position in the virtual environment.

The magnetic sensory ability is obtained by adding vibration feedback for interactions with magnetic fields and virtual objects. The operator, wearing the data glove, extends their hand toward the magnet and engages in gestural interactions at varying distances from the magnet. Concurrently, the output of the PFES varies with both distance and gesture, as depicted in Fig. 5e. The strain-magnetic field signal from a single sensor cannot be directly used to characterize the distance information from the magnet due to the influence of gestures and finger movements.

Based on the DRL-KD method, models can be quickly deployed to analyze the time series data of the 5 PFES units in the data glove, predict the distance information between the hand and the magnet, and control the vibration signal. The divergence of the probability distribution is used to directly match the data probability distribution between the teacher and student feature spaces rather than the softmax function and distillation temperature parameters in gesture classification work^59^. The cosine similarity of the output feature space is used to replace the classification label to measure the difference between the teacher and student model distributions. To minimize the difference in the probability distribution, the Kullback–Leibler (KL) divergence is defined as the loss function for training the model as ${{\mathscr{L}}}_{{\rm{KL}}}=\sum P\cdot \log P/Q$, where $P$ and $Q$ are the probability distributions of the teacher model and student model, respectively. The distillation loss ${{\mathscr{L}}}_{{\rm{DL}}}$ and ground-truth loss ${{\mathscr{L}}}_{{\rm{CE}}}$ are defined consistent with those in the previous text, and the root mean square error (RMSE) is used in the prediction task to replace the cross-entropy loss function in the classification task.

The draw-wire encoder is used to measure the actual distance between the palm and the magnet. The model structure and participants of the teacher model, assistant model, and student model remain unchanged, and the output data of gesture interactions at different distances from the magnet are collected. The output data of the 5 PFES units are tiled into a 1-dimensional time series of length 20, and the teacher model and assistant model are trained and tested. Figure 5f shows that compared with traditional deep learning networks, the DRL-KD model can deploy regression models for new users to predict hand positions. The traditional three-layer LSTM model requires more than 70 training epochs to reach a normalized RMSE of 0.015. The model extracted using our developed learning framework shows a normalized RMSE of less than 0.005 within 50 transfer training epochs.

Figure 5g shows the comparison between the actual measured value of the distance between the user’s hand and the magnet and the predicted value of the student model output. Some deviations in the predicted values occur at the moment of gesture interaction, but the position changes with the magnet can be clearly distinguished. After the distance information is debounced and filtered, the noise can be effectively reduced, and it can be mapped to a 50–100% pulse duty cycle to control the output of the LRA vibrators. The vibration instances of the LRA vibrators are visually characterized by piezoelectric vibration sensors. The vibration intensity of all LRAs synchronously increases when approaching a virtual object, allowing feedback of information from the virtual scene to the operator, as shown in Fig. 5h. By manipulating virtual objects using the PFES and magnet in the real world, the data glove builds motion maps from the human hand and magnet to the virtual hand and object. Through this simplified natural interaction platform and feedback system, we successfully obtained magnetic perception capabilities and simulated interactive operations with virtual objects.

## Discussion

Based on the sensitivity of magnetostrictive films in the magnetized state to external strain or magnetic fields, we propose a PFES with a bioinspired hierarchical structure for measuring the curvature radius and magnetic field to determine the fusion of shape and position. The electrodeposition method combined with MEMS technology can achieve the preparation of PFES with a minimum thickness of 120 µm and arbitrary bending deformation characteristics. The experiment verified that the maximum sensitivity of the PFES was 22 mV/mm for a curvature radius ranging from 10 to 65 mm and 1.78 mV/(kA/m) for a magnetic field ranging from 1 to 11 kA/m. In addition, the PFES exhibited a fast response (17 ms) and recovery (39 ms) time. The changes in the sensitivity are less than 1.6% under different *ρ* and *H* values at 1–4 Hz, which indicates good dynamic output stability.

To demonstrate the perceptive fusion performance of the PFES, we designed a data glove system with autonomous learning to be applied to human-computer interaction scenarios. The DRL-KD method is developed for compressing teacher models by the autonomous learning of models and their knowledge, which is closer to the user’s motion characteristics, and can quickly deploy student models in wearable devices and accurately comprehend the user’s motion characteristics. The data glove system with DRL-KD has the ability to add new gestures through different models and can obtain up to 88.06% accuracy for confusing gestures related to body position, which is difficult for traditional single-mode sensors and their interactive interfaces. The developed learning framework is used for regression of the magnetic field perceived by the PFES to extract the hand position information as the control signal of haptic feedback, which can provide the operator with magnetic perception and virtual target interaction capabilities, with a standardized RMSE of less than 0.005. The PFES combined with autonomous learning can fuse the shape and position dual information of the finger joints, ultimately achieving human‒machine interaction with gesture recognition and haptic feedback for cross-space perception and manipulation.

## Materials and methods

### Test platform loading to the curvature radius and magnetic field

An experimental apparatus was set up that can provide an adjustable curvature radius *ρ* and a magnetic field *H* to test the performance of the PFES. A 3-point bend fixture with a dynamic mechanical analyzer (DN50, DANA) was used to determine the loading curvature radius. The magnetic field was loaded with a permanent magnet (NdFeB N35), and the magnetic field intensity was calibrated with a Hall effect Gaussian meter (8030, F.W. Bell). The fixture is placed along the axial direction of the PFES to simultaneously load the radius of curvature and the magnetic field. The MI layer of the PFES is linked to a waveform generator that outputs an alternating current as the excitation source. The output voltage of the pick-up coils is amplified by a voltage preamplifier (SR560, Stanford Research Systems) and then analyzed by monitoring the data acquired by the PC.

### Gesture recognition experiments and dataset collection

To correct the output differences of the PFES caused by different wearing habits and finger sizes of the users, a standardization process was adopted during the establishment of the dataset, calibrating the data glove with the maximum and minimum output signal values of the PFES. The fingers were straightened, the palm was flattened, and the palm was placed 5 cm away from the magnetic marker in front of the chest to calibrate the minimum output value of the PFES. The fingers were clenched to maintain a fist shape, making the fingers reach the maximum bending state, to calibrate the maximum output value of the PFES. When the hand is close to the magnetic marker on the chest, finger bending movements are difficult, and the output signal drops sharply. The signals output within the range less than 5 cm from the magnetic marker in front of the chest are considered the minimum output of the PFES.

After calibration, the data glove can be used to monitor gesture data sending the data to the host computer whose interface is based on the MATLAB GUI. The computer maps the signal to a range from 0 to 1. When the data glove captures gesture motion information, the user’s unconscious random jitter and the signal acquisition amplification process will produce random high-frequency noise. The amplitude-limiting jitter filtering method is used to process the data collected by the data glove, which has a good filtering effect for slowly changing measured parameters.

The active segment data are segmented from the filtered signal for calculation and recognition by the classification model. When the hand is in a resting state or a moving state, the amplitude of the PFES signal has obvious differences, which can be used to determine the start and end points of the effective signal of the gesture action collected by the data glove, that is, the start and end points of the active segment. When there is no action, the PFES signal fluctuates stably and slightly. The larger the action is, the greater the fluctuation. The active segment can be segmented according to the data variance. A moving window is used to frame the normalized multichannel data, and the variance of each frame is calculated as $S=\frac{1}{M}{\sum }_{M}\frac{1}{N}{\sum }_{N}{\left[{X}_{M}\left(N\right)-\bar{{X}_{M}(N)}\right]}^{2}$, where${X}_{M}(N)$ represents the signal sequence of the data frame in any channel, N is the length of the data frame, and M is the channel number of the PFES.

The total variance *S* is calculated through the time series data of 5 channels. In the data transmission window, when *S* is greater than $1* {10}^{-3}$, the starting point of the gesture signal is determined. Within 1–10 s after the start point, a time point with $S < 1* {10}^{-3}$ is searched for as the end point. If a time point greater than $1* {10}^{-3}$ is not found within the range of 1–10 s later, this starting point is considered random jitter; this point is discarded, and the next starting point is searched.

### Supplementary information

the PFES output data acquisition for different gestures

Pytorch-based DRL-KD programs and data sets collected by the PFES
